# ‘I no longer know that person’: Experiences of families living with someone using crystal methamphetamine

**DOI:** 10.1371/journal.pone.0284156

**Published:** 2023-04-12

**Authors:** Dara Sampson, Milena Heinsch, Jenny Geddes, Richard Velleman, Gill Velleman, Maree Teesson, Nicola Newton, Frances Kay-Lambkin

**Affiliations:** 1 School of Medicine and Public Health, University of Newcastle, Newcastle, Australia; 2 National Health and Medical Research Centre for Mental Health and Substance Use, University of New South Wales, Sydney, Australia; 3 Department of Psychology, University of Bath, Bath, United Kingdom; 4 Velleman Consultancy Ltd, Bath, United Kingdom; 5 Addictions Research Group, Sangath Foundation, Goa, India; Stellenbosch University, SOUTH AFRICA

## Abstract

**Background:**

Crystal methamphetamine (‘ice’) use is a large and growing worldwide problem, yet few research studies have explored the impact of crystal methamphetamine use on affected friends and family members. We explored the experiences and narratives of family members and friends of people who use methamphetamine to inform the development of a Family and Friend Support Program.

**Methods:**

This paper reports on a subset of findings from a mixed method study, which sought to better understand the experiences of family members and friends of people who use methamphetamine. Participants were recruited via Facebook advertising and asked to complete a survey outlining their experiences. At the end of the survey, participants were invited to be interviewed by a clinical psychologist on the research team, to discuss their experiences in greater depth; seventeen people agreed to be interviewed. This paper is based on a qualitative thematic analysis of these interviews using Braun and Clarke’s 6-stage approach to identify key areas of concern for friends and family members of people who use methamphetamine.

**Results:**

Through thematic analysis, five key themes were developed, namely: (1) loss, (2) stigma, (3) support (or lack thereof), (4) ways of coping, and (5) the value in sharing personal experiences. The results of this study revealed the profound sadness, frustration and loss friends and family members experienced when caring for a loved one who uses methamphetamine. This loss was further complicated by societal stigma surrounding the use of methamphetamine, which often extended to friends and family members themselves. Since experiences of grief and loss were interwoven across the three themes, concepts of ambiguous loss, disenfranchised grief, and narrative constructivist approaches to understanding loss, were applied to the discussion of results.

**Conclusion:**

This study provides a more complete picture of family and caregiver experiences when caring for a loved one using methamphetamine, which may further help inform the design of intervention programs. Implications for future research and practice with this population are considered.

## Introduction

Methamphetamine use has become a major public health concern, with many countries reporting increased use and harms in recent years [1]. Global estimates show that between 5.4 and 9.8 million people were amphetamine dependent in 2017, with the highest prevalence recorded in Australasia and North America [2]. Regular methamphetamine use is associated with increased mortality [3], as well as cardiovascular and immune diseases and severe neurological complications such as incidences of psychosis and major depressive disorder [4–7]. This has significant consequences for public health, especially in light of findings that emergency hospitalisations following methamphetamine use are higher than for any other drug [8].

Methamphetamine use can also have devastating impacts on the wellbeing of caregivers [9,10]. Families providing support to a person with a substance use disorder often experience considerable strain, which can negatively impact many areas of their life, including their own health and wellbeing [11–13]. For this reason, families and friends affected by substance use disorders are increasingly viewed as a group who require support and assistance in their own right [14]. Findings from two decades of qualitative research highlight the adverse impacts of general substance use on families, especially on parents, spouses, and children [15].

Despite rising societal concern about ice, relatively little is known about the impact of a person’s ice use on their family and friends. There have been studies done on the impacts for children living with a parent who uses methamphetamine [16,17], however their focus, whilst equally valuable, was in the child protection arena, although one does touch upon the impact on grandparents [17]. A broader-based Australian quantitative study captured research priority areas for methamphetamine and emerging drugs in Australia, however of the four priority areas identified, the needs of family and friends did not rank [18]. In particular, there is a lack of qualitative research exploring the experiences and coping strategies of friends and family members in managing, specifically, the psychosocial consequences of caring for a loved one using methamphetamine. Our recent scoping review which, after de-duplication sourced 2257 records, identified only four qualitative accounts of caregiver experiences and coping and two evaluation studies examining interventions for family members of people who use methamphetamine [19]. Findings from these studies revealed the elevated levels of stress experienced by caregivers, both as a consequence of their loved one’s mental health decline and problematic behaviours, and as a consequence of their own feelings of distress, grief, fear, guilt and shame [20]. They also highlighted the interpersonal conflicts these tensions can give rise to across family and friendship groups [21]. Findings from these studies highlight the need for further qualitative research as opposed to quantitative research, in order to more richly explore the complex experiences and concerns of friends and family members caring for a loved one using methamphetamine.

In this paper, we report on a subset of qualitative findings from a mixed method study, which sought to better understand the experiences of friends and family members affected by a loved one’s methamphetamine use. While this study did not explicitly set out to examine the grief and loss of friends and family members caring for a loved one using methamphetamine, these experiences were found to permeate across all themes identified in this study. A decision was, therefore, made to apply contemporary grief and loss theories to the discussion of findings. To date, there has not been specific consideration of the grief and loss friends and family members can experience when someone they are close to uses methamphetamine. This paper, thus, extends the existing literature in this area.

## Conceptualising grief and loss

There is currently limited literature exploring the grief and loss experiences of friends and family members who inhabit the world of the person using alcohol/other drugs. Existing literature focuses either on the grief processes of the person using alcohol/other drugs [22] or on families’ experiences of grief when a person using drugs dies [23]. The intangible grief that can occur while a person using drugs is still present requires further examination.

Historically, grief and loss was conceptualised primarily as a response to death [24]; traditional grief and loss theorists promoted detachment from the deceased, and investment in new relationships [25,26]. Contemporary theorists challenge the notion that there is one ‘right’ way of grieving and are more interested in understanding *how* people grieve [27–30]. Further, constructivist theorists have a keen interest in exploring people’s personal stories, or ‘narratives’, of loss and the distinct meanings they draw from this experience, which are neither objective nor defined [31–33]. The degree to which this loss confirms or challenges a person’s worldview is believed to influence their grief response. For example, the death of an older person may be more congruent with a person’s expectations of loss than the loss of a child.

There has also been increased interest in grief resulting from different forms of loss [34,35], based on recognition that change itself (be it positive or negative) can bring with it a sense of loss [34]. Change may take the form of shifts in employment [35], relationships, identity, future plans, social status or development, amongst many others. Because the losses associated with these changes are often less tangible, their impact may be underestimated; the individual experiencing the loss, and those around them, may minimise or de-value the experience. Two specific conceptualisations of loss are particularly pertinent to this experience—ambiguous loss and disenfranchised grief.

Ambiguous loss occurs when one element of a person remains, while another is lost [36]. A person may either be physically lost but psychologically present (for example a person who has been reported ‘missing’), or physically present but psychologically ‘lost’ to friends and relatives (for instance, a person with dementia, mental illness, cognitive impairment, or substance use disorders). This form of loss can be particularly challenging due to its amorphous nature—the person physically remains in the lives of those they know but is no longer ‘known’. Disenfranchised grief locates loss within social constructs [37–40] that create an evaluation of the relative ‘worth’ of a loss and determine what forms of loss are socially acceptable to grieve [38]. In this sense, the person experiencing the loss may be denied ‘permission’ (at an often-unconscious level) to experience their loss due to the perception held culturally of the ‘worthiness’ of that grief.

Culturally, in Australia at least, there is a reticence to discuss issues of grief and loss. It is still seen by many people as a taboo subject [41]; often as a result of fear of ‘saying the wrong thing’ which can lead to avoidance of deep discussion and provision of support [42].

## Methods

### Study design and recruitment

The findings reported in this paper are situated within a wider mixed method study with friends and family members of people who use methamphetamine. Participants were recruited via Facebook advertising between February and March 2017. The study included a brief questionnaire—the Short Questionnaire for Family Members (Affected by Addiction) (SQFM(AA)), a 33-item questionnaire based on the stress-strain-coping-support (SSCS) model of addiction and the family [15,43]—followed by a number of open-ended questions which were used to gain insight into the experiences of affected family members and friends. Participants who completed the survey were invited to take part in a follow-up semi-structured qualitative interview via telephone to discuss their experiences in more detail. Although limitations exist in telephone interviews, such as rapport-building challenges and lack of non-verbal cues, these were acknowledged and mitigated by allowing up to 90 minutes for interviews which would be conducted by an experienced clinical psychologist. This paper reports the findings from the telephone interviews. This research was approved by the University of Newcastle Human Research Ethics Committee; H-2017-0040. Active, written and verbal (audio recorded) informed consent was provided by all project participants directly. No minors were eligible for participation.

### Participants

A total of 39 participants completed the survey. Of these, 24 agreed to be contacted for an interview. However, 7 participants could not be contacted within the study period, therefore 17 telephone interviews were completed. We deemed that a sample size of 17 was sufficient to reach data saturation using the information power model [44] developed by Malterud and colleagues, which considers study aim, sample specificity, theoretical background, quality of dialogue, and strategy for analysis. Our study aim and sample were narrow and specific, exploring the experiences of friends and family members affected by a loved one’s methamphetamine use; we used well-established grief and loss theories in our interpretation of the research findings; the interview content was rich and deep; and we conducted an in-depth thematic analysis. Of the 17 participants, nine were mothers of people using methamphetamine. The remaining eight participants were siblings, fiancés, husbands, brothers, flat-mates, or in-laws. Those participants who agreed to a telephone interview were offered a $20 voucher to a music/games store.

### Interview protocol and procedure

The interviews were conducted between February and April 2017 and comprised the qualitative component of the study. They were informed by interpretative phenomenological research principles which value story and the perspective of those at the centre of their story. This approach also guided the data analysis [45,46]. Orford and colleagues’ [47] approach provided a frame for the telephone interviews. Interviews were semi-structured and focused on a range of areas including people’s experience of supporting a friend or family member using crystal methamphetamine (challenges, triumphs, journey), coping strategies, resilience factors, sources of support (for them directly, and their loved one) and gaps/barriers to accessing assistance. Interviews ranged from 60 to 90 minutes and were conducted by a clinical psychologist, who was part of the research team. While the interviews involved structured components, where possible, a free-flowing narrative was encouraged, as many participants had not had the opportunity to talk about their experiences of supporting someone using methamphetamine before, and since the richness of client experience is best elicited without constraints [48,49]. There were 18 questions in total, however five were deemed to be key questions, in that they tended to elicit answers to other questions, thus creating the opportunity for a free-flowing conversation. These questions are outlined in Table 1 (note: the ‘X’ represents the name of the participant’s family member, or friend, using crystal methamphetamine).

**Table 1:** Interview questions.

Question number	Questions
**Information**	
1.	Some people who are affected by another person’s use of alcohol/other drugs have said that they would like more information about alcohol/other drug addiction and its effects. Is this true for you?
2.	What other information (apart from information about alcohol/other drug addiction) might be/have been helpful for you?
**Coping**	
3.	How have you have reacted or attempted to cope with X’s alcohol/drug use?
**Support**	
4.	Who helped you during this difficult time?
**Program development**	
5.	What do you think about a support program aimed at helping you, a family member/friend affected by X’s use of ‘ice’, as opposed to help aimed at X?

### Data analysis

Interviews were transcribed verbatim using OutScribe, an online transcription service. Data were analysed by the research team using Braun and Clarke’s 6-stage reflexive thematic analysis approach [50–53]. The application of reflexive thematic analysis meant our analysis could be both inductive, staying close to and driven by the data, while also allowing theoretical flexibility for grief and loss theories to inform later the discussion of the analysis. The lead author began by familiarising themselves with the data by reading and re-reading the transcripts. The researcher then engaged in a process of open coding, attributing code labels to segments of data. Codes were discussed with the research team and a set of themes were constructed. In keeping with reflexive thematic analysis principles, the collaborative process was crucial not in seeking consensus, but in deepening the interpretation of the data. During this process, the importance that participants attributed to a behaviour, belief or concept was considered paramount. Finally, five key themes were developed from our analysis: loss, stigma, support (or lack thereof), ways of coping, and the value in sharing personal experiences.

## Results

Friends and family members in this study described experiencing significant and extreme changes to their lifestyle, working life and relationships, as well as increased psychological and financial distress. Participants described their experiences of being close to someone using methamphetamine as ‘stressful’, ‘chaotic’, and ‘unpredictable’, often resulting from verbal abuse, physical aggression, and violence. Friends and family members reported variable levels of support for their current situation. While some had the support of other family members and friends, many others reporting experiences of social isolation. Inaccurate portrayals of methamphetamine use which generate negative stereotypes, and the experience of stigma were common. Five themes were identified in the data. The preeminent theme was that of loss. The following four themes are highly relevant as they intersect with, and contribute to, the loss experienced by friends and family members.

### Loss of affected person and identity of carer

The experience of loss emerged as a strong presence throughout friends and family members’ stories, despite not forming part of any formal questioning. It was the pre-eminent theme, in itself, and overlapped the other identified themes. In telling their stories, friends and family members regularly described experiences of loss permeating through all aspects of their lives.

The experience of ‘loss’ covered many domains for friends and family members in the current study and was present both in discussions about the person using methamphetamine, and when the friends and family member discussed themselves. Loss included actual physical loss via death:

Participant 1 [woman who had lost her daughter to suicide via overdose]: *“I don’t understand what it did to her to help her … and take herself out”*

Participant 1: *“But we all go*, *we all have battles that we need to*, *and*, *you know*, *as I say to myself and other people when they say that*, *you know*, *the loss of your child*. *But as I say*, *I had mine for 21 years*. *Some people don’t have them for 21 minutes”*

Participant 1: *“I’ve had a definite change in who I am through the loss of a child”*.

‘Loss’ also permeated friends and family members’ stories through their descriptions of the person they once knew, someone who had changed, a known and loved person who had become seemingly lost to methamphetamine use:

Participant 3: *“She was great*, *professional*, *wouldn’t swear*, *wouldn’t lie*, *wouldn’t steal*, *you know perfect member of society*, *very professional*… *the opposite*. (Later after starting using methamphetamine) *Would steal*, *would lie*, *swear like a trooper*, *you know*. *Basically*, *living on the streets*.*”*

Also reported, was a loss of future dreams and goals, both for friends and family members and for their loved one using methamphetamine.

Participant 4: *“We have got*, *um*, *her four children*, *ah*, *in permanent care with us*, *now*. *Um*, *when I say us*, *me and my husband*. *Ah*, *but in the last six months*, *me and my husband have split up*. *And it’s all been caused through this…um*, *as well as me having a nervous breakdown*. *And*, *yeah*, *its*, *its um*, *it’s just*, *yeah*, *and I’m a mot-*, *ah*, *I’m a grandmother of um*, *21 grandkids*, *so…these four grandkids are*, *are taking over our lives*, *you know*? *And I carry a lot of resentment for the kids… And that’s not fair*. *I know it’s not their fault*. *But I just resent them so much… for taking this part of our life away”*

Participant 4: *“And that* [money stolen] *was going to go on buying an RV and we were going to travel around Australia”*

Grief and loss extended to aspirations as to what the loved one could have been, including concerns about their future ability to lead a normal life.

Participant 5: *“From the very beginning I’ve worked with him to keep a life and build a life*. *I made sure he*, *during that time*, *he finished an apprenticeship*. *I drove him to work for four years*, *and I lived out of town*, *to make sure he got work”*

Participant 6: *“And that broke*, *it broke my heart*. *Like I just cried because I was like*, *you worked so hard and that was something that was just yours*. *And no one else had anything to do with it*. *And that was something you could have been proud of*. *And you’ve lost it all*.*”*

(Girlfriend of person using methamphetamine who had lost his DJ business due to drug use)

For some friends and family members in our study, the loss experienced was reflective, not a fully formed response; a sense of no longer *‘knowing’* the person using methamphetamine.

Participant 1: *“I couldn’t believe that this was my child and I thought… what have you done with my child*? *Can I please have her back please*?*”*

Participant 1: *“This isn’t the person I know”*.

Others spoke of observing a separation between the former *‘person’* and their current persona:

Participant 5: *“I’d see him… where you’d look and you’d go*, *oh he’s gone*. *He’s gone*, *he’s gone*…*”*

Participant 1: *“I think the thing that I have mostly taken from it is I thought I knew my child…*. *There’s a child in there and then there’s a drug addict”*.

Participants spoke of enormous disruption to, and loss of, a ‘normal’ life. This was reflected in comments about social disruption, as well as at very practical levels. The role of caring for someone impacted by methamphetamine became paramount in people’s daily lives:

Participant 7: *“Because this became a full-time job”*

Participant 8: *“Because I am sitting here all day with no motivation*. *I’m just smoking continuously*, *one after the other”*.

Participant 8: *“Nothing*. *Been trying to get me haircut for two months and no*, *that’s just out of the question*, *at the moment*, *as well”*.

The experience of loss extended to family cohesion, with many friends and family members highlighting the devastating impact of methamphetamine use on family relationships. For some, the loss of relationships and family cohesion also further limited the support they could receive from those family members, thereby isolating them further.

Participant 8: *“Everybody’s divided*. *Everybody is just divided*. *I argue with them all the time*, *they tell me to um*, *stop trying to help her*. *I have ruined the family by allowing her to keep coming here and trying to help her*.*”*

The impact of methamphetamine use on families was pronounced, destroying and dividing families:

Participant 4: *“It’s just destroyed the whole family…it’s drawn a wedge between me and my other kids”*

As well as the loss of one’s drug using relative, either because they had changed so substantially or through death, and the loss of the hopes and aspirations that the participants had for that relative, and for their hoped-for lives together, there was also a loss of self, highlighted in the ways friends and family members described how they had altered as a person, as a result of living with someone who was using methamphetamine. This included both loss of self to psychological distress, as well as the loss of self, resultant from disruption to life roles and activities.

Participant 8: *“I’m a changed person”*

Participant 6: *“And so… I was lost*. *I couldn’t actually help myself because all of my energy and all of my emotions were going into try and get him help”*

Participant 4: *“Well I gave up my nursing when we got the* [grand]*kids…I’ve given up working for other things… we lost our friends”*

The loss reported by friends and family members was not linear and interviewees found it challenging to ‘resolve’ because it was also influenced by the nuance of hope and cycles of despair.

Participant 8: *“You’ll have her out*, *she’ll be back here in a couple of days*, *and we’ll be dealing with the same shit”*

Participant 5: *“You’re feeling despair*, *and there are no words that are going to comfort a mother’s heart from that despair”*

Respondents often reflected on this cycle with comments such as:

Participant 9: “*This isn’t my first ride on the merry-go-round”*.

Participant 8: *“And I always get the same thing*. *Always “I’m not going to do it anymore”*. *It’s ruined my life*. *I just want my family back*. *I just want my son back”*

Capacity to find cognitive congruence was often difficult for the friends and family member participants. One participant stated:

Participant 3: *‘‘I don’t know how people live with this*, *I really don’t*.*”*

Another participant spoke of being trapped, proclaiming:

Participant 4: *“There is no escape”*.

Being ‘lost’ was a particular word which arose on a multitude of occasions. For most participants it was incredibly difficult to fathom why and how they had, not only lost a sense of the person they love but lost themselves in the process.

Participant 4: *“And now because they are in our care*, *I can’t have my husband to myself*. *It’s just that I’m so lonely…”*

Trying to find meaning was an on-going, complex process.

Participant 2: *“I just stopped looking for the reasons why and um*, *I don’t know*. *There’s no point in agonizing over why it’s happened*. *You’ve just got to go*, *well its happened*. *And what do we do*?*”*

In summary, participants spoke of family breakdown, changes in family dynamics, feeling dislocated from family, friends and society, and the loss of the person using methamphetamine, as well as the loss of their own sense of ‘self’; all associated with their loved one’s use of methamphetamine. There was evidence of complex emotions ranging from despair (participant 7“…*because we were just over it*”) to fear (Participant 8“*I did put a lock on my door last year when she was threatening me*”) to guilt (Participant 1“…*and I’ll never forgive myself”*).

This led to an experience of living with or supporting a person using methamphetamine that was characterised by guilt, shame, and stigma. The impact of experiencing loss that could not be openly acknowledged was also significant.

### The experience of stigma for this particular substance usage

The experience of stigma on affected family members and friends was evident throughout many stories, with friends and family members frequently reporting social isolation as a result. This further extended to include discussions about the stigma of methamphetamine use and the difficulties of public grieving.

Participant 7: *“What I found out very early on is that you can’t trust anyone… you have to be very selective on who knows about this… extremely selective*. *Because people are very judging*.*”*

Friends and family members reflected that stigma, whether directly experienced, perceived, or feared, affected their inclination to seek support from others, including professionals, social networks, and, indeed, sometimes within their own family unit:

Participant 1: *“That’s probably my stigma because the way I see it is I’ve already lost my child… do I have to go through more*?*”*

Participant 2: *“I kept it from my friends… I don’t know that I’ve even really admitted to them that he’s a drug addict… and I think I’ve disguised his bizarre behaviour in terms of the mental health issues”*

Friends and family members suggested that the stigma of methamphetamine use added to their isolation and, therefore, inhibited the grieving process. One participant encapsulated this by saying:

Participant1: *“Because I still think there’s such a stigma attached to saying out loud my son or daughter uses ice*. *I don’t say it out loud”*.

Fear of social disapprobation meant sadness was often internalised. One participant described this with the metaphor:

Participant 9: *‘‘Oh*, *I lock them* [feelings] *away in that little box*, *you know*, *that no-one ever talks about*, *and I don’t think about it*.*”*

### Systemic lack of support

Participants spoke of a systemic lack of support. This ranged from friends and family to mental health services and police. Many associated this with negative societal perceptions of the relative worth of people using methamphetamine, highlighting a link between lack of support and stigma:

Participant 8: *“I rang the police station; they told me to ring the MST team*. *I rang the MST team; they told me to ring somewhere else*. *I rang somewhere else; they told me it was the police’s responsibility*. *I rang back the police*, *they told me it wasn’t their responsibility*. *Thirty phone calls later…*. *Nobody was responsible”*

Participant 10: *“Ice addicts come into accident and emergency*, *and you see things like that*, *and they’re horrible despicable people*, *but the person loves them doesn’t think that way*, *and sometimes they just need support from other people who are in the situation*. *They’re not bad people and someone does care about how they are feeling”*

### Ways of coping: Strategies for survival

Participants described the ways in which they tried to ‘cope’ with the impact of their loved one using methamphetamine. Some strategies were practical (e.g., information garnering). Others involved talking to someone (although complicated by responses of some people and value judgements). Several participants described seeking ‘comfort’ through eating, exercise or watching television. Still others spoke of a need to write and reflect. Participants often ignored their own needs to prioritise those of the person with the addiction.

For some participants time and prioritising self were challenging.

Participant 11: *“He thinks I should do counselling but*, *um*, *it’s just trying to fit it in*.*”*

Participant 7: *“So*, *your health*, *your mental health*, *your physical health*, *your emotional health*, *becomes*, *just goes further and further down the list… and the same with all your other family members*. *So*, *because the problem is so consuming that it just covers everything else”*

Participant 12: *“I’ve tried to help my brother to the point that it’s been to my own detriment*.*”*

On a positive note, one participant named belief as a means of surviving.

Participant 13: “How have I coped? Because I’ve always had hope.”

### The value in sharing personal experiences

Participants spoke of a desire to generate support for other people in a similar situation through the sharing of their story.

Participant 1: *“When this tragedy happened*, *we all looked at each other and went*, *yeah this can shatter families*. *This won’t shatter our family*. *There’s got to be something good that comes out of a tragedy”*

This was a highly motivating reason for participation and far exceeded people’s interest in a tangible gain. (Participants were offered a music store voucher—some declined, and others chose to give the voucher to someone else.) Parallel to this altruistic motivation was a valuing in being able to tell their story in a non-judgemental climate.

Participant 14: *“…if this can make it easier for someone else*.*”*

## Discussion

We explored the experiences of family and friends caring for a loved one using methamphetamine. Seventeen interviews were conducted, which revealed the profound sadness, frustration and loss friends and family members can experience when caring for a loved one who uses methamphetamine; the compounding impacts of societal constructions of drug use on caregivers’ experiences of loss; and the strategies caregivers use to cope with these impacts. Since experiences of grief and loss were intertwined with all themes identified in this study, a decision was made to apply contemporary grief and loss theories to the discussion of findings.

‘Loss’ was present throughout the stories friends and family members told about their loved one; the person they once knew. Participants spoke of the loss of their hopes and aspirations in relation to what their loved one might have achieved. They described their own loss of self-identity and the future they had planned or hoped to have. Many attested to feeling ongoing love and affection for their family member or friend, but identified that the person had changed, perhaps irrevocably. For some, methamphetamine was an entity in itself, separate from their loved one’s identity. This finding is consistent with ambiguous loss [36]; the person using methamphetamine was physically present, but their family and friends either felt psychologically estranged from the person or had made a conscious choice to separate the former relationship from the current one. The experiences of loss described by participants are similar to those reported in the broader caregiving literature, which highlights the loss of identity [54,55] and sense of grief caregivers can experience when integral and unique aspects of their connection with their loved one are lost [56,57]. These findings affirm the importance of providing support and assistance to family members and friends in their own right, and not solely to the person using methamphetamine [14].

Findings from this study also revealed the additional burden experienced by caregivers of people using methamphetamine. Participants in this study had to manage their own, often complex experiences—sometimes hopeful and sometimes despairing—of change. At the same time, they had to contend with the judgements and expectations of others, not only in relation to the use of drugs and the ‘type’ of people who use them, but in relation to families and friends who support people with a substance use disorder. From a theoretical perspective, their grief was therefore rendered ‘disenfranchised’ [27–29]; the loss was not readily perceived as such, either by participants, or by the people in their immediate social circles, making it difficult for them to share their pain and feelings of grief with others. For many participants, this led to feelings of isolation, even from their closest friends and family, from whom they would normally draw support. This finding supports previous qualitative research, which identified the interpersonal conflicts experienced by friends and family members caring for a person using methamphetamine [4–7]. It is especially concerning in light of research highlighting the important role of family relationships, bonding and connectedness as a protective factor and source of resilience [58], particularly during times of crisis [59]. Future interventions must address the complexities families and friends experience in managing their grief whilst contending with societal expectations about how (and even if) they should grieve.

Participants in this study demonstrated a strong commitment to telling their story, describing experiences and events that led to a myriad of emotions—dislocation, bewilderment, anger, anguish, and denial. Key to this ‘storytelling’ process was a quest to try to ‘make sense’ of these experiences and create a narrative (and sometimes counter-narrative). Having their narrative heard and validated was important to participants, and many expressed a sense of hope that their story would support and help others in their meaning-making journey. Along with this sense of altruism was a consciousness of the nature of storytelling as a healing process—a desire to place their individual experience in a broader context. This use of storytelling and language to make sense of the world is consistent with the constructivist notion of ‘meaning making’ [31–33] in that participants were able to find some meaning in their loss through the process of recounting their story. It supports findings from the broader literature about the transformative and healing power of story and storytelling for people experiencing grief and loss [60,61]. This is particularly important in light of the finding from this study that talking to someone is a coping strategy for caregivers of people using methamphetamine. It presents an encouraging opportunity for future research to design narrative interventions for friends and family members of people who use methamphetamine; an endeavour that is particularly urgent given the limited interventions specifically designed for this population [19].

## Limitations and implications for future research and practice

While the sample size for this research is relatively small, the strength of the qualitative approach in providing in-depth data balances out this issue. Findings from this study provided important insights into participants’ complex experiences of grief and loss in relation to their friend or family member using methamphetamine. This suggests that an area for further research and clinical consideration is the idea that ambiguous loss, combined with societal disenfranchisement, hinders meaning making. Participants were dealing with major changes which were made more complex by managing the stigma attached to their grief. These societal constructions, and the cycles of hope and despair described by participants, rendered the grief process less defined and visible. The grief described by participants in this study was one which ‘cycled’ between a sense of loss and a sense of hope. Adopting this ‘cyclic’ framework would provide an integrated foundation for public health care workers who are supporting people caring for a loved one using methamphetamine.

## Conclusions

The loss of a loved one is life changing. The world is never quite as it was. Participants in this project unreservedly shared their stories of sadness, frustration, and loss. Application of grief and loss theory revealed that their experiences were emblematic of ambiguous and disenfranchised grief, and about trying to make meaning of the changes in their loved one. Participants living with or supporting someone using methamphetamine spoke of their fear of social judgement, their feelings of blame and guilt, and their lack of ‘logical’ explanation as to why they were continuing to support a person with this particular addiction. Service providers can support friends and family members experiencing these challenges by allowing them the opportunity to share their story. Grief can be a difficult discussion topic. This research serves as a reminder of the importance of story, active listening, and advanced empathy, when working with people caring for a loved one using methamphetamine.
